# Study on the Attenuation of Long-Term Skid Resistance of Asphalt Mixtures under Aeolian Sand Conditions

**DOI:** 10.3390/ma16237247

**Published:** 2023-11-21

**Authors:** Jingsheng Pan, Ping Li, Liu Zhao, Qiang Pan, Jiaming Ni, Yong Wang

**Affiliations:** 1 Department of Civil Engineering, Chengdu Technological University, Yibin 644000, China; pjsheng1@cdtu.edu.cn (J.P.); lping2@cdtu.edu.cn (P.L.); zliu1@cdtu.edu.cn (L.Z.); njming1@cdtu.edu.cn (J.N.); 2 College of Water and Architectural Engineering, Shihezi University, Shihezi 832003, China; wyong@shzu.edu.cn

**Keywords:** asphalt pavement, aeolian sand, skid resistance, accelerated abrasion, gray correlation analysis

## Abstract

In this work, the long-term skid resistance attenuation law of asphalt mixtures in the presence of aeolian sand was studied. Four types of asphalt mixtures underwent skid resistance abrasion tests using an accelerated loading tester. The pendulum value (BPN) and structure depth (MTD) of these four mixtures were determined under various conditions of sand density and abrasion times. The correlation between the BPN and density and the number of times of abrasion were investigated, respectively, to analyze the skid resistance attenuation law at the microscopic and macroscopic levels. Our results indicate that the skid resistance of the four types of asphalt mixtures initially decreased and subsequently reached a stable state. Sand density primarily influences skid resistance during the initial stage, while the number of abrasions becomes the dominant factor affecting skid resistance in the later stages.

## 1. Introduction

In different stages of use of asphalt pavement, the road surface should always maintain good skid resistance to provide sufficient friction between the car wheels and the road surface. With the increase in traffic volume, the attenuation trend of asphalt pavement skid resistance is becoming increasingly prominent. Insufficient road skid resistance is one of the reasons for traffic accidents [1]. Therefore, improving road skid resistance is an important guarantee for driving safety.

The road surface texture is a key factor in determining the skid resistance of pavements; good road surface macro-construction and micro-texture is crucial to improve the skid resistance, and the asphalt mixture gradation type, coarse and fine aggregate lithology, and other factors also affect the skid resistance of pavements [2,3,4,5,6,7]. However, in reality, the road surface texture is inevitably affected by external environmental factors, such as rainfall, snowfall, icing, and other pollutants, and the presence of these adherents affects the friction between vehicle tires and the road surface [8,9]. Changes in climate and traffic volume have a significant impact on the pavement texture [10,11,12]; rainfall seriously affects the anti-skid performance of the pavement [13,14,15]; and the rainwater present becomes a lubricant between the tire and the pavement, with the water film producing dynamic water pressure on the wheels, so that the tire adhesion to the pavement decreases as the thickness of the film increases, leading to a greater decline in the anti-skid performance [16,17]. Under freezing conditions, ice and snow fill the road surface voids and reduce the depth of the structure, hindering the direct contact between the tires and the road surface, and the anti-skid performance is then seriously reduced [7,18,19,20], resulting in the loss of the original stability of the vehicle braking and an increase in the braking distance, which poses a potential hazard to driving safety. There are other pollutants on the road surface, such as sand, salt, and oil leakage, which will also negatively affect the anti-skid performance of the road [21,22] over a long period of time; as the wheels roll over, these pollutants on the road surface are diffused, leading to compaction, thus blocking the asphalt pavement gaps and reducing the roughness of the road surface, so that the road surface loses its original anti-skid performance, especially in the presence of wind-accumulated sand. In addition, changes in temperature also affect the anti-skid performance of pavements [23,24], and the anti-skid performance in summer is generally lower than that in winter under the conditions of a clean road surface. All of the above are problems faced by asphalt pavement skid resistance performance, the pavement skid resistance performance is vulnerable to the interference of the external environment.

Although many studies have been carried out on the anti-skid performance of asphalt pavements, these studies mainly focus on the study of anti-skid performance in the absence of abrasion and the sensitivity analysis of the influencing factors, especially the study of the anti-skid performance of pavements under the conditions of the presence of attachments, which are not able to capture the abrasion of the pavement texture via an attachment under the action of the wheels. In desert areas, wind and sand are particularly harmful to roads [25,26]. Among them, wind sand covering the road surface is one of the typical hazards, and research has shown that the contact area in a certain proportion will have an impact on the anti-skid performance [27]; the wind accumulation of sand reduces the effective contact area between the tires and the road surface, in which the larger particles cover the surface of the road, hindering the direct contact between the tires and the road surface; smaller particles fill the macroscopic structure of the road surface and reduce the coefficient of friction, which makes the asphalt pavement anti-skid performance seriously degraded, and the accumulation of sand becomes an important reason for the use of a weakened road surface function.

In this paper, we will combine the actual situation of sand accumulation on desert highways, analyze the morphological characteristics of aeolian sand, use accelerated loading abrasion equipment to simulate the skid resistance of asphalt pavement under aeolian sand conditions over its whole life cycle, and analyze the trend of texture change in asphalt pavements and the evolution of skid resistance under different amounts of sand accumulation. The results of the study are expected to provide a reference for the prevention and control of desert highway diseases, especially in terms of skid resistance.

## 2. Materials and Methods

### 2.1. Test Materials

The aggregate selected in this study is sandstone from Xinjiang, China. According to the “Aggregate Testing Procedure for Highway Engineering” [JTG E42-2005 (Ministry of Transportation of the People’s Republic of China)] [28], technical indexes such as abrasive value, crushing value, and abrasion value of coarse aggregate were tested, and the angular properties of fine aggregate were tested via the flow method. The test results all meet the requirements, as shown in Table 1.

Table 2 shows the gradation types AC-16, SMA-13, and SMA-16. The aggregates are all sandstone with the same specific gravity, so the pass rate of each stage is controlled by quality. The pass rate of each sieve is shown in Table 2. The optimal oil stone ratios are as follows: AC-13 is 4.7%, AC-16 is 4.2%, SMA-13 is 6.1%, and SMA-16 is 5.9%.

**Figure 1:**
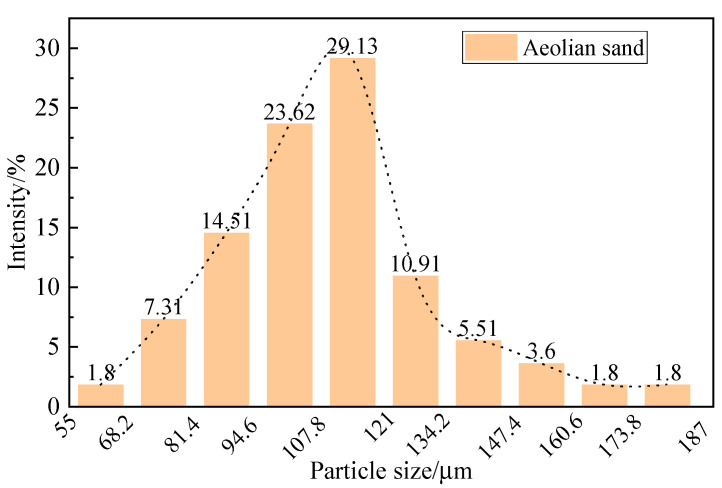
Results of the grain size analysis of wind-accumulated sand.

**Figure 2:**
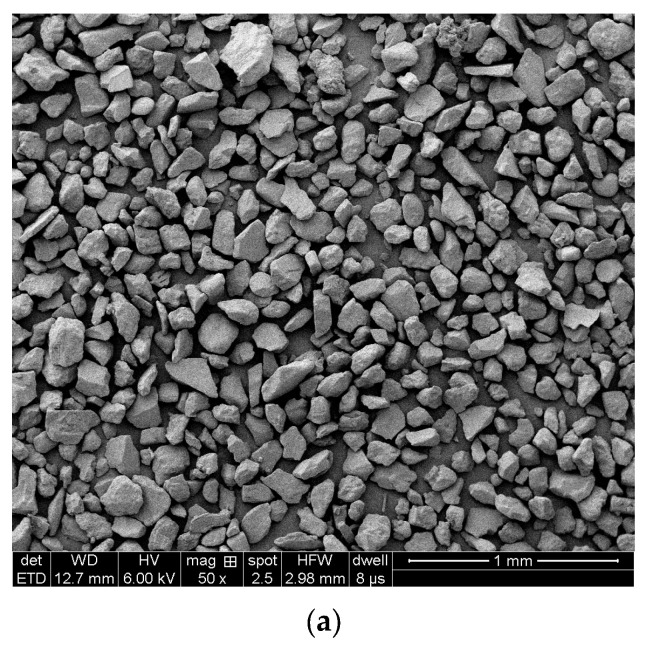

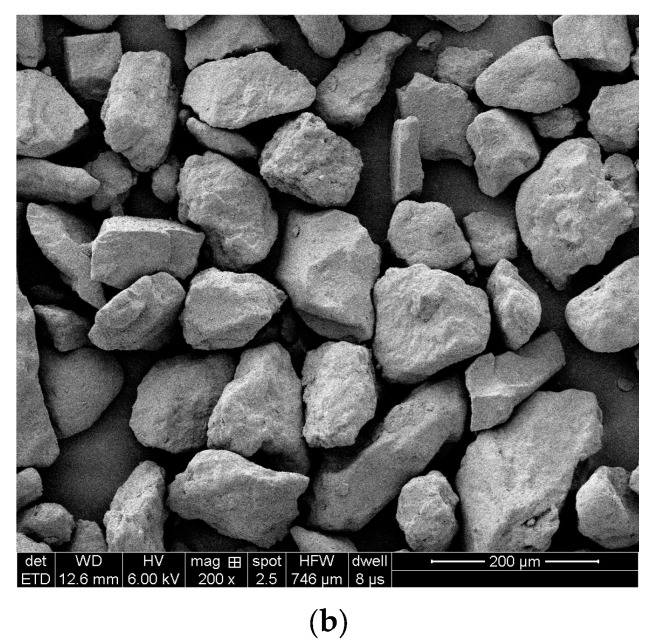
Aeolian sand morphology at (**a**) 50X and (**b**) 200X magnitude.

Wind-accumulated sand is the accumulated sand covering the surface of the highway in the desert area of Xinjiang. The particle size distribution of the samples was tested using a laser particle size analyzer (instrument model: Mastersizer 2000), and the results are shown in Figure 1, and the morphology of the samples of aeolian sand was obtained using a field emission scanning electron microscope (FEG250), as shown in Figure 2.

The results of the laser particle size analyzer showed that the particle size of the aeolian sand was between 55 and 187 μm, which was mainly distributed between 94.6 and 121 μm, and the percentage of this part of the particle size was 53.7%. Figure 2 shows the morphology of windlogged sand particles under a scanning electron microscope (SEM), and the granular shape is uniform.

### 2.2. Test Methods

Four types of asphalt mixtures, AC-13, AC-16, SMA-13, and SMA-16 were prepared. Based on the accelerated abrasion equipment, accelerated abrasion tests were carried out on four types of asphalt mixtures with different numbers of test specimens to test the BPN and MTD of specimens with different sand densities after a certain number of abrasions; the dimensions of test specimens were 300 mm × 300 mm × 50 mm, the BPN values were obtained from a pendulum friction coefficient tester, and all the tests were carried out at room temperature.

## 3. Results and Discussion

### 3.1. Skid Resistance Performance

**Figure 3:**
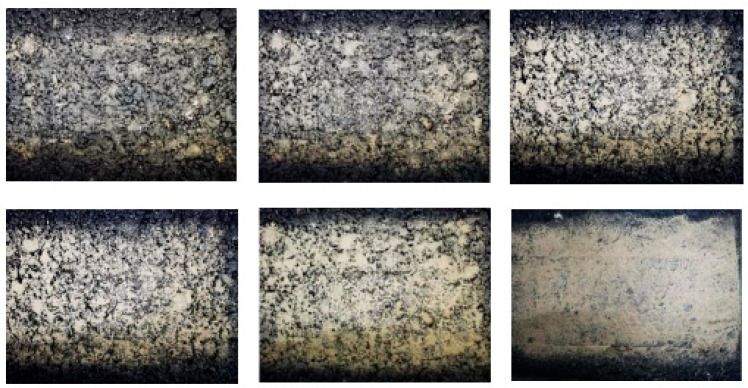
State of the specimen under different aeolian sand deposition densities.

**Figure 4:**
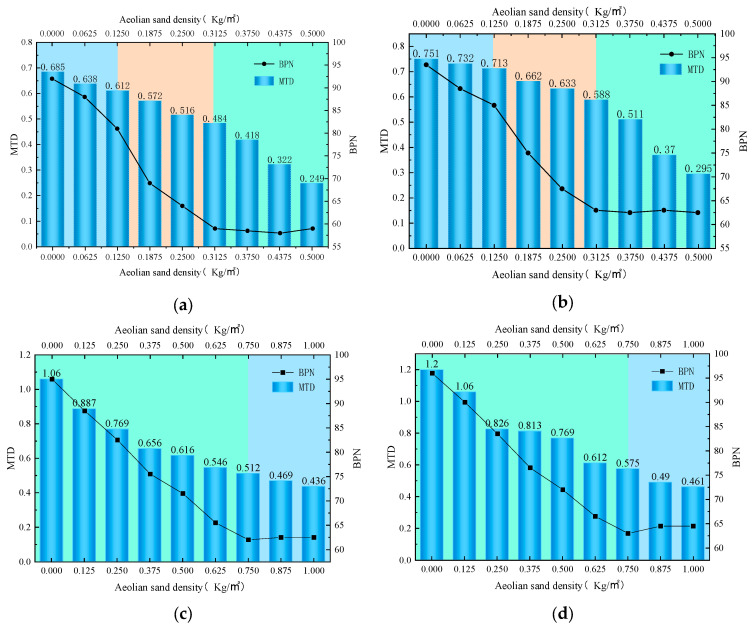
BPN and MTD at different sand accumulation densities. (**a**) AC-13, (**b**) AC-16, (**c**) SMA-13, (**d**) SMA-16.

In order to investigate the effect of sand density on the skid resistance of different pavement structures, four types of asphalt mixture specimens were produced to simulate the actual sand conditions of the road surface according to different sand densities, as shown in Figure 3. The variation patterns of BPN and MTD under different sand density are shown in Figure 4.

Shown in Figure 4 are the change in trend of BPN and MTD values of different types of asphalt mixture specimens under different sand density conditions. As a whole, the change rule of BPN and MTD values of four types of asphalt mixture specimens under different sand density conditions is basically the same, which decreases with the increase in sand density.

Figure 4a,b show the change patterns of AC-13 and AC-16 asphalt mixtures, respectively. Among them, the change in BPN value can be divided into three stages, which are marked with three colors of background in Figure 4, blue represents the first stage, orange represents the second stage, and green represents the third stage. The process of increasing the sand density from 0 to 0.125 kg/m^2^ is the first stage, in which the BPN value decreases slowly, the BPN value of AC-13 decreases by 12.0%, the BPN value of AC-16 decreases by 9.1%, and the decrease in the BPN value of AC-13 is much larger than that of AC-16, and the second stage is the process of increasing the sand density from 0.125 kg/m^2^ to 0.3125 kg/m^2^. The third stage is the process of increasing the density of sand accumulation from 0.3125 kg/m^2^ to 0.5 kg/m^2^; at this time, the curve gradually becomes flat, the BPN value tends to stabilize, and it can be regarded as the critical point of the decay of the BPN value when the density of sand accumulation is 0.3125 kg/m^2^. The change in the MTD value can be divided into two phases, and the process of the increase in the density of sand accumulation from 0 to 0.3125 kg/m^2^ is the first phase; at this time, the MTD value decreases with the increase in the density of sand accumulation, in which the MTD value of the AC-13 decreases by 39.0%. The MTD value of AC-13 decreased by 39.0%, and the MTD value of AC-16 decreased by 32.0%, and the decrease in the value of AC-13 was larger than that of AC-16. When the sand density increased from 0.3125 kg/m^2^ to 0.5 kg/m^2^, the process was the second stage, and the decrease in the MTD value became larger in this stage, and the two kinds of asphalt mixtures decreased by 40.4% and 42.3%, respectively.

Figure 4c,d show the change patterns of SMA-13 and SMA-16 asphalt mixtures, respectively. Among them, the change in BPN value can be divided into two stages, which are marked with two colors of background in the Figure 4, green represents the first stage, and blue represents the second stage. The process of sand accumulation density increasing from 0 to 0.75 kg/m^2^ is the first stage, in which the BPN value decreases slowly, the BPN value of SMA-13 decreases by 34.7%, the BPN value of SMA-16 decreases by 34.4%, and the decrease in the BPN value of SMA-13 is slightly larger than that of SMA-16, and the second stage is the process of sand accumulation density increasing from 0.75 kg/m^2^ to 1.0 kg/m^2^, in which the BPN value tends to stabilize, and it can be considered that the sand density of 0.750 kg/m^2^ is the critical point for the decay of the BPN value. The change in the MTD value can be divided into three stages, and the process of the increase in the sand density from 0 to 0.125 kg/m^2^ is the first stage, in which the MTD value decreases with the increase in the sand density, in which the MTD value of SMA-13 decreases by 16.3%, SMA-13 decreases by 16.3%, SMA-16 MTD decreases by 11.7%, SMA-13 decreases by more than SMA-16; when the sand density increased from 0.3125 kg/m^2^ to 0.5 kg/m^2^ process for the second stage, in this stage, the MTD value decreased more, the two kinds of asphalt mixtures decreased by 34.4% and 27.5%, respectively, and in the third stage, it tends to stabilize.

It is analyzed that, due to the aeolian sand particles’ dense, small particle size, it is easy for them to enter the asphalt mixture specimen in the gap, thus filling part of the gap, making the MTD value lower, leading to anti-skid performance, and then with the increase in the density of sand accumulation, asphalt mixture gaps are gradually filled; at this time, the MTD value of the rapid decline in the BPN value also sees a rapid decline. When part of the asphalt mixture surface was covered in wind-accumulated sand, as shown in Figure 3, the wheel–sand–pavement trio form a micro-bearing system, changing the sliding friction for rolling friction, and reducing the anti-skid performance dramatically; when the sand density is 0.3125 kg/m^2^ (AC-13, AC-16) and 0.750 kg/m^2^ (SMA-13, SMA-16) for the critical point, at this time, the BPN value tends to stabilize, and the friction is controlled by the sand accumulation, which is caused by the shortcomings of the pendulum friction coefficient meter, and with the increase in sand density, as shown in Figure 3, the wind sand produces resistance to the rubber slider, which makes the BPN value rebound, and this phenomenon is caused by the shortcomings of the experimental apparatus. However, in practice, the aeolian sand coverage is large enough to produce congestion on the wheels, resulting in an increase in vehicle resistance.

### 3.2. Accelerated Wear Test

**Figure 5:**
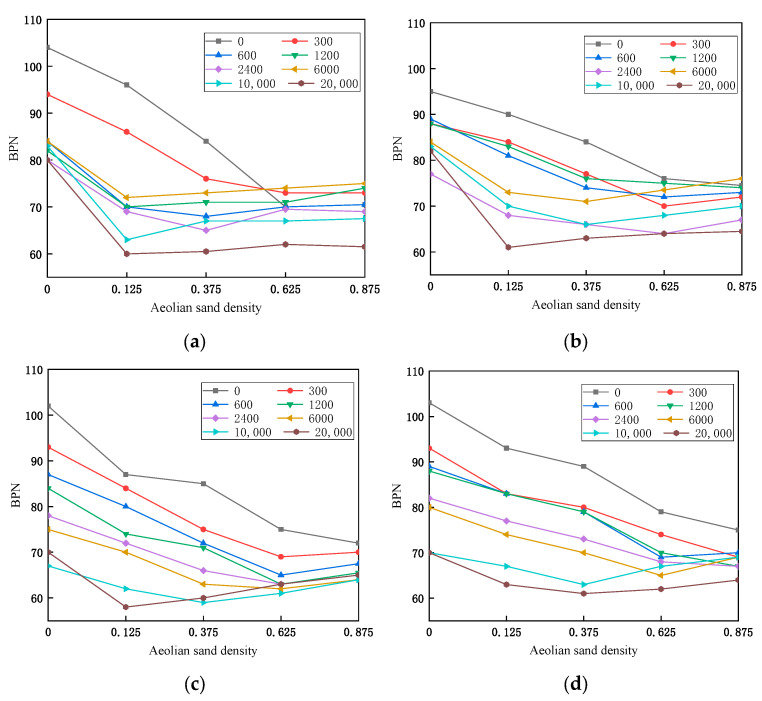
BPN decay under different amounts of sand accumulation. (**a**) AC-13, (**b**) AC-16, (**c**) SMA-13, (**d**) SMA-16.

In order to study the effect of wheel and sand accumulation on asphalt pavement skid resistance, under different sand density conditions, accelerated abrasion equipment is used to accelerate the abrasion of asphalt mixture specimens for different times, which simulates the real situation of the wheel on the sand accumulation on the pavement, and then measures the BPN value, and the results of the test are shown in Figure 5.

As shown in Figure 5, overall, four types of asphalt mixture specimens were produced in the pre-abrasion period: the SMA-graded type of mixture anti-skid performance is better than the AC-graded type of asphalt mixture, under the action of the wind accumulation of sand; with the increase in the number of times of abrasion, the anti-skid performance has shown a decreasing trend, when the density of sand accumulation is smaller, the BPN value decreases significantly. Moreover, after the number of abrasions reaches 10,000 times, with the increase in sand accumulation, the BPN value shows a small increase after decreasing to a certain degree.

Figure 5a,b show the change rule of the BPN value of the AC-graded asphalt mixture specimens under different abrasion times and sand density. For AC-13, when the number of abrasions is more than 300 times, the sand density is less than 0.125 kg/m^2^; when the BPN value decreases rapidly and when the sand density is more than 0.125 kg/m^2^, the BPN value tends to stabilize. It is observed that the AC-13-graded asphalt mixture porosity and structural depth is small, and after a certain number of abrasions, the structural depth and voids are reduced, the specimen surface can accommodate the sand inside, the space becomes smaller, the sand is more likely to cover the surface of the specimen, and then the change in BPN tends toward a stable state. For AC-16, when the number of abrasions is less than 1200 times, the change in BPN value is similar to that of AC-13. It is analyzed that the depth of structure and porosity of AC-16 are larger than those of AC-13, so it needs more times of abrasion. Therefore, for the AC-graded asphalt mixtures, 0.125 kg/m^2^ is the inflection point of the trend of the BPN value.

Figure 5c,d show the change rule of the BPN value of the SMA-graded asphalt mixture specimens under different abrasion times and sand density. For SMA-13, the BPN value is not stabilized when the sand density is 0.125 kg/m^2^, and the BPN value tends to be stabilized when the number of abrasions is not more than 6000 times and the sand density is 0.625 kg/m^2^. The BPN value of SMA-16 has the same rule of change, but the BPN value still does not show a stabilizing tendency when the number of abrasion is not more than 300 times and the sand density is 0.875 kg/m^2^, and this is the reason why the BPN value does not show a stabilizing tendency. The value still does not show a stabilizing trend, which is due to the fact that SMA-16 has larger voids and tectonic depths relative to SMA-13.

The aeolian sand covers the asphalt pavement, and after repeated rolling by the wheels, the macroscopic structure of the road surface is directly affected, resulting in significant changes in the road surface texture, leading to a decrease in the anti-slip performance of the road surface. Therefore, it is believed that the wear of the wheels on the road surface can reduce the anti-slip performance of the road surface. It is possible to study the impact of wear on the anti-slip performance of asphalt pavement under the presence of wind-blown sand by studying the changes in BPN values under different wear times and sand density conditions.

**Figure 6:**
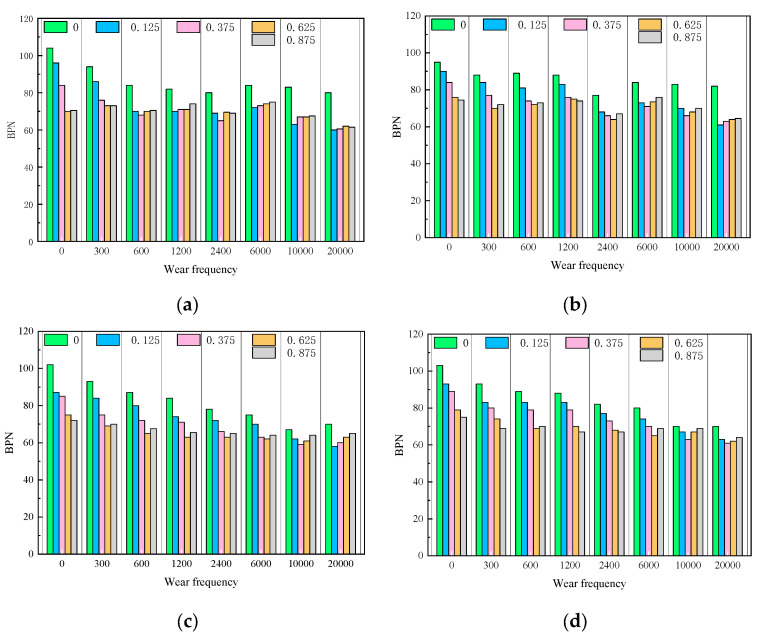
BPN decay with different number of abrasions. (**a**) AC-13, (**b**) AC-16, (**c**) SMA-13, (**d**) SMA-16.

Shown in Figure 6 are the four grades of asphalt mixtures in the sand state under different abrasion times under the BPN value of the change rule. In the same number of abrasions, with the increase in sand density, the BPN value first decreases and then tends to stabilize; if the number of abrasions is small, the BPN value has a significant downward trend, and when the number of abrasions increased to a certain value, the sub-precipitation of the presence of sand under the conditions of the BPN value fell to the minimum, and then tends to stabilize the state. This phenomenon exists in both the AC-graded and SMA-graded asphalt mixtures. For AC-13, in the first 300 times of abrasion, the density of sand accumulation can affect the skid resistance performance, and after 300 times of abrasion, the BPN value almost does not change with the increase in the density of sand accumulation, as shown in Figure 6a. It is analyzed that this is due to the number of abrasions after 300 times, wherein the AC-13 specimen surface texture is almost destroyed, the depth of the structure is reduced, the surface does not accommodate the gap in sand accumulation, and at this time, the wind sand can only be piled up on the surface of the specimen; when the density increases a little bit, the BPN value tends to be stabilized, and even if the density of sand accumulation increases, the BPN value does not change significantly when the density of sand accumulation is increased to 0.625–0.875 kg/m^2^, the BPN value is not changed significantly when the density of sand accumulation increases to 0.625–0.875 kg/m^2^, and there appears to be a small increase in the BPN value during the phenomenon. Since the void ratio and structural depth of AC-16 are slightly larger than that of AC-13, the BPN value is almost unaffected by the sand density after the number of abrasion reaches 1200 times.

This law also exists in the SMA-graded asphalt mixtures. Due to the relationship between the void ratio and the depth of the structure, the BPN value does not decrease with the increase in sand density after the number of abrasions of the SMA-graded asphalt mixtures reaches 2400 times, as shown in Figure 6c,d.

### 3.3. Gray Correlation Analysis

**Figure 7:**
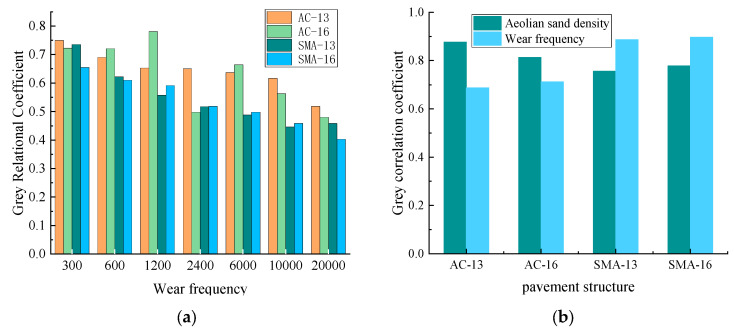
Results of gray correlation analysis (**a**) the sensitivity analysis of asphalt pavement skid resistance performance by different abrasion times (**b**) the factor sensitivity analysis of the number of abrasion and sand density on the anti-skid performance of asphalt pavement.

In order to determine the degree of influence of the number of abrasions and the density of sand accumulation on the anti-skid performance of asphalt pavement, the test results were subjected to gray correlation analysis, and the results are shown in Figure 7. Figure 7a shows the sensitivity analysis of asphalt pavement skid resistance performance according to different abrasion times; for AC-13, SMA-13, and SMA-16, the gray correlation coefficient is the largest when the number of abrasion times is 300 times, which means that the abrasion in the early stage of the asphalt pavement skid resistance performance is the largest; in reality, this is usually in the early stage of the road being open to traffic and the skid resistance performance decreases faster; this phenomenon is consistent with the test results. However, the gray correlation coefficient of AC-16 is the largest when the number of abrasions is 1200 times, which means that the impact on the anti-skid performance of the pavement is the largest when the number of abrasions is increased to 1200 times. Figure 7b shows the factor sensitivity analysis of the number of abrasion and sand density on the anti-skid performance of asphalt pavement; for AC-13 and AC-16, the gray correlation coefficient of sand density is larger than that of the number of abrasions, which implies that the sand density has a greater influence on AC-13 and AC-16 than the number of abrasions. However, for SMA-13 and SMA-16, the gray correlation coefficient of the number of abrasions is greater than the sand density. It was analyzed that because the macro structure and internal void of the SMA-graded asphalt mixture are larger than that of the AC-graded asphalt mixture, and this void can accommodate part of the sand, when the internal void is not completely filled, the density of sand will not have a significant effect on the anti-slip performance, while the number of abrasion directly destroys the macroscopic and microscopic structure of the pavement, so the number of abrasions has more influence on the anti-slip performance than the density of sand for the SMA-graded asphalt mixture. Therefore, for SMA-graded asphalt mixtures, the number of abrasions has a greater effect on skid resistance than the sand density. For AC-graded asphalt mixtures, the sand density has a more direct effect on the skid resistance due to the smaller depth of the structure and the internal voids.

## 4. Conclusions

In this paper, based on aeolian sand, using a special pavement attachment, the effect on the skid resistance of asphalt pavements under the conditions of the presence of sub-deposited sand is investigated. This is a study of changes in skid resistance based on macro- and micro-perspectives caused by the effect of different sand accumulation densities, abrasion times, and asphalt mixture gradation types, and the following conclusions are obtained based on the experimental study and data analysis:

1. In the presence of wind-accumulated sand, the BPN values of AC-graded asphalt mixtures show a three-stage decreasing trend, and the MTD values show a two-stage decreasing trend; the BPN values and MTD values of the SMA-graded asphalt mixtures both show a two-stage decreasing trend.
2. The accelerated loading abrasion test was used to simulate the change in anti-skid performance of asphalt mixtures under aeolian sand conditions under the action of vehicle loading, and the results showed that the decline in anti-skid performance was accelerated after the abrasion of aeolian sandy pavements, the density of sand accumulation was the main factor affecting the anti-skid performance in the early stage, and the number of abrasion times was the main factor affecting the anti-skid performance in the later stages.
3. With the help of gray correlation analysis to establish the correlation ranking between each factor and the asphalt mixture anti-skid performance, with regard to the AC-graded asphalt mixture, sand density is the main factor affecting the anti-skid performance; regarding the SMA-graded asphalt mixture, the number of times of abrasion is the main factor affecting the anti-skid performance; for two grades of asphalt mixture, a value of 300 times of abrasion is the main factor affecting the anti-skid performance. In order to improve the long-term skid resistance of an aeolian sand asphalt pavement, it is recommended to use asphalt mixtures with a larger aggregate particle size and larger void ratio.

The author’s future research prospects include jointly considering the effects of traffic volume, temperature, and aeolian sand in a laboratory-scale environment with a wider range of conditions, and evaluating the skid resistance of an aeolian sand asphalt pavement.

## Figures and Tables

**Table 1:** Aggregate performance.

Testing Program	Test Results	Technical Indicators
Crushing value (%)	12.6	≤26
Wear value (%)	10.3	≤28
Polished stone value (PSV)	39	≥36
Angularity (s)	31.9	≥30

**Table 2:** Gradation of each asphalt mixture.

	Percentage Passing through Each Sieve Opening										
Gradation types	19	16	13.2	9.5	4.75	2.36	1.18	0.6	0.3	0.15	0.075
AC-13	100	100	95.1	77.8	51.6	25.4	16.2	12.7	8.4	6.9	5.5
AC-16	100	95.6	84.8	69.6	49.4	35.6	24.7	19.7	11.9	8.6	5.0
SMA-13	100	100	95	63	27	21	19	16	13	12	10
SMA-16	100	95	75	55	26	19.5	18	15	12.5	11.5	10

## Data Availability

Data are contained within the article.
